# The Business of Charity: Remarkable Examples

**Published:** 1913-12-27

**Authors:** 


### THE BUSINESS OF CHARITY: REMARKABLE EXAMPLES.

Our issue this week is occupied almost entirely with matters which relate directly to the business of charity. We give an account of the proceedings at the meeting of the constituents of the Hospital Sunday Fund under its new constitution, which shows that whereas the receipts on Hospital Sunday 1913 have been relatively small the improvement in the business management of this fund has attracted to the Mansion House a larger number of representatives of all the congregations than have come in the ordinary course to these meetings for twenty years past. That is a hopeful sign. It shows that the people have not only not lost interest in Hospital Sunday, but that they believe in it, and that the real underlying cause of the diminished yield from this fund lies on the laps of the clergy and ministers of all denominations throughout the Metropolis. The speeches, reported elsewhere, have gone straight to the point, and we hope we may hear less of the groans of religious leaders over empty churches and chapels and find more evidence of the awakening of these leaders in regard to Hospital Sunday and to the needs of the people, for whose consolation, uplifting, and protection these places of worship have been created and built. What the Hospital Sunday Fund needs is an added sense of responsibility with a knowledge of the glories of personal service throughout the whole religious community of the Metropolis of the Empire. Many years ago at the request of the clergy, a percentage of the total collections was set aside to supply medical and surgical appliances, convalescent letters, and other valuable aids to the work of a Christian minister. One of those who has had to do with the Sunday Fund for many years past writes with conviction that many of the heads of congregations have come to look upon the Sunday Fund too exclusively as a centre of supplies. Many seem to have forgotten it is the privilege of every minister himself to realise the responsibility of giving such an output of personal service every year as shall secure that every member of his congregation shall contribute, at any rate something on Hospital Sunday, towards the support of our hospitals. Wake up, clergy and ministers, for then your congregations will arouse themselves too, and Hospital Sunday in London may yet raise the £100,000 a year it can readily do.

The report of the proceedings at a special meeting of the governors of St. George's Hospital should be studied with care by all who are associated with the administration or who take an interest in the work of the greater hospitals. It affords evidence that there is something wrong with St. George's Hospital. What that wrong is due to was made plainly apparent at Friday's meeting. St. George's Hospital occupies the best site of any hospital in London. It should undoubtedly be the easiest of all hospitals to raise money for. Yet the figures show that the ordinary income of St. George's Hospital has so drooped for twenty years that it has only increased by 5 per cent. on the total in 1891. Moreover, the ordinary income during this twenty years of the Middlesex Hospital has increased by 25 per cent., of University College Hospital by 76 per cent., of Charing Cross Hospital by 57 per cent., and of the Royal Free Hospital by 94 per cent. These figures are convincing, but their force is driven home with telling effect by the knowledge that the London Hospital, which has to raise an enormous sum every year by voluntary contributions, has increased its ordinary income by no less a sum than 125 per cent. in the last twenty years, largely due to the driving force supplied by Mr. Sydney Holland.

The League of Mercy, through its chief officials and some thousands of members, presidents, and vice-presidents, in fourteen years has raised in small sums and handed to the hospitals approximately £220,000. The keynote, spirit, and motive of the League of Mercy is personal service by each individual in the days of health in the cause of the sick. Its constituents represent all classes. From the circumstance that it raises its revenue in small sums, it has keenly felt directly the effects of compulsory National Insurance. 1913 has drooped indeed, but there is a certain stern resolution amongst those responsible for raising voluntary funds, which revealed itself at St. James's Palace, where hearty co-operation and the belief in the doctrine of personal service, created an atmosphere which stirred the heart. The League of Mercy needs 600 more men and women of good will, who are willing to devote, in the whole, some three days a year each to personal service in the cause of the hospitals. We mention this because we believe there must be amongst the readers of the The Hospital a great many quiet and purposeful people of both sexes, who would like to give a little of themselves to such a cause, deliberately, regularly, and with all their might. If our belief is well founded, will each one of them send his or her name and address to the Editor, some time between now and December 31 next?

